# ISG15 deficiency restricts HIV-1 infection

**DOI:** 10.1371/journal.ppat.1010405

**Published:** 2022-03-25

**Authors:** Denise Jurczyszak, Lara Manganaro, Sofija Buta, Conor Gruber, Marta Martin-Fernandez, Justin Taft, Roosheel S. Patel, Melissa Cipolla, Hala Alshammary, Lubbertus C. F. Mulder, Ravi Sachidanandam, Dusan Bogunovic, Viviana Simon

**Affiliations:** 1 Department of Microbiology, Icahn School of Medicine at Mount Sinai, New York City, New York, United States of America; 2 Center for Inborn Errors of Immunity, Icahn School of Medicine at Mount Sinai, New York city, New York, United States of America; 3 Precision Immunology Institute, Icahn School of Medicine at Mount Sinai, New York City, New York, United States of America; 4 Mindich Child Health and Development Institute, Icahn School of Medicine at Mount Sinai, New York City, New York, United States of America; 5 Department of Pediatrics, Icahn School of Medicine at Mount Sinai, New York city, New York, United States of America; 6 Department of Oncological Sciences, Icahn School of Medicine at Mount Sinai, New York City, New York, United States of America; 7 INGM-Istituto Nazionale di Genetica Molecolare, Virology, Milan, Italy; 8 Department of Pharmacological and Biomolecular Sciences (DiSFeB), University of MIlan, Milan, Italy; 9 Global Health and Emerging Pathogens Institute, Icahn School of Medicine at Mount Sinai, New York City, New York, United States of America; 10 Division of Infectious Diseases, Department of Medicine, Icahn School of Medicine at Mount Sinai, New York City, New York, United States of America; 11 Department of Pathology, Molecular and Cell-Based Medicine, Icahn School of Medicine at Mount Sinai, New York City, New York, United States of America; University of Wisconsin, UNITED STATES

## Abstract

Type I interferons (IFN-Is) are a group of potent inflammatory and antiviral cytokines. They induce IFN stimulated genes (ISGs), which act as proinflammatory mediators, antiviral effectors, and negative regulators of the IFN-I signaling cascade itself. One such regulator is interferon stimulated gene 15 (ISG15). Humans with complete ISG15 deficiency express persistently elevated levels of ISGs, and consequently, exhibit broad spectrum resistance to viral infection. Here, we demonstrate that IFN-I primed fibroblasts derived from ISG15-deficient individuals are more resistant to infection with single-cycle HIV-1 compared to healthy control fibroblasts. Complementation with both wild-type (WT) ISG15 and ISG15ΔGG (incapable of ISGylation while retaining negative regulation activity) was sufficient to reverse this phenotype, restoring susceptibility to infection to levels comparable to WT cells. Furthermore, CRISPR-edited ISG15^ko^ primary CD4^+^ T cells were less susceptible to HIV-1 infection compared to cells treated with non-targeting controls. Transcriptome analysis of these CRISPR-edited ISG15^ko^ primary CD4^+^ T cells recapitulated the ISG signatures of ISG15 deficient patients. Taken together, we document that the increased broad-spectrum viral resistance in ISG15-deficiency also extends to HIV-1 and is driven by a combination of T-cell-specific ISGs, with both known and unknown functions, predicted to target HIV-1 replication at multiple steps.

## Author summary

Type I interferons (IFN-Is) are a group of potent inflammatory and antiviral agents. They induce IFN stimulated genes (ISGs), which perform downstream functions to resolve viral infection, mediate the inflammatory response, as well as negatively regulate the IFN-I signaling cascade to prevent hyperinflammation. One such negative regulator is interferon stimulated gene 15 (ISG15). Humans that lack ISG15 have chronic, low levels of antiviral ISGs, and ensuing broad-spectrum resistance to viral infection. We demonstrate that IFN-I priming of ISG15-deficient cells leads to superior resistance to human immunodeficiency virus 1 (HIV-1) infection compared to IFN-I primed healthy control cells. This is true for fibroblast cell lines, as well as primary CD4+ T cells, the main target of HIV-1. Analysis of the gene expression profiles show that ISG15-knockout CD4^+^ T cells express similar inflammatory markers as ISG15-deficient patients. Overall, we show that the broad-spectrum viral resistance in ISG15-deficiency extends to HIV-1.

## Introduction

Type I interferons (IFN-Is) are the first line of defense against viral infections. IFN-I signaling induces the Janus kinase (JAK)–signal transducer and activator of transcription (STAT) pathway, leading to transcription of about 400 IFN-I-stimulated genes (ISGs) [1]. These ISGs act as antiviral effectors, but some also function as negative regulators of IFN-I signaling in order to prevent overt inflammation. One such negative regulator of IFN-I signaling is interferon stimulated gene 15 (ISG15). ISG15 exists as a free intracellular molecule, free extracellular cytokine, and as a ubiquitin-like conjugate through a process called ISGylation [2–5]. Free intracellular ISG15 aids the stabilization of the ubiquitin-specific peptidase 18 (USP18), which downregulates IFN-I signaling by binding to the IFN-I receptor subunit, IFNAR2, and out-competing JAK1 [6]. Thus, a loss of ISG15 leads to the destabilization of USP18, leading to continued IFN-I signaling, and ensuing IFN-I mediated autoinflammation [7]. Free extracellular ISG15 serves to induce IFN-γ in natural killer (NK) and T cells, in synergy with IL-12. Individuals with complete ISG15 deficiency are thus also more susceptible to Mycobacterial infections [8]. Finally, although ISGylation has been reported to alter the activities and functions of many proteins, the exact role for ISGylation is still debated [9–24].

There are strong differences among species in the role for ISG15 in viral immunity, as unlike human ISG15 which serves to stabilize USP18, the murine orthologue does not perform such activity [25]. Thereby, mice lacking ISG15 were shown to be modestly more susceptible to some but not all viruses [11,26–33], while humans with ISG15 deficiency have overactive antiviral responses and no documented susceptibility to viral infections [6–8,25,34–36]. In turn, these patients suffer from clinical sequalae of chronic inflammation marked by increased levels of ISGs [6–8,34]. To recapitulate the persistent IFN-I signaling observed in ISG15-deficient patients *in vivo*, we have optimized an *in vitro* protocol which permits testing of viral susceptibility in a physiologically-relevant cell culture model [6]. Replication of RNA and DNA viruses such as VSV, HSV-1, Influenza A virus, Sendai virus, Nipah virus, and Rift Valley Fever virus was attenuated in ISG15-deficient, IFN-I-primed cells as compared to healthy control IFN-I-primed cells [6]. These data suggest that ISG15-deficiency may actually provide for increased resistance to severe viral infections.

IFN-I signaling and the role of ISG15 in the context of HIV-1 infection is poorly understood. As expected, IFN-I stimulation has been shown to inhibit HIV-1 replication, due to the induction of several ISGs that function as general antivirals [37–52]. Among these, ISGylation of Gag was demonstrated to inhibit HIV-1 budding [11,53–55]. Yet, more recently, a proviral role for USP18 in primary human macrophages was described [56,57]. Given these conflicting data regarding the HIV-1-specific antiviral or proviral effects of ISG15, we sought to investigate the role of ISG15 in regulating susceptibility to HIV-1 infection. Specifically, we investigated these functions under the physiological low-level expression of ISGs observed in ISG15-deficiency.

## Results

### ISG15 deficient, IFN-primed cells are less susceptible to HIV-1 infection

**Fig 1:**
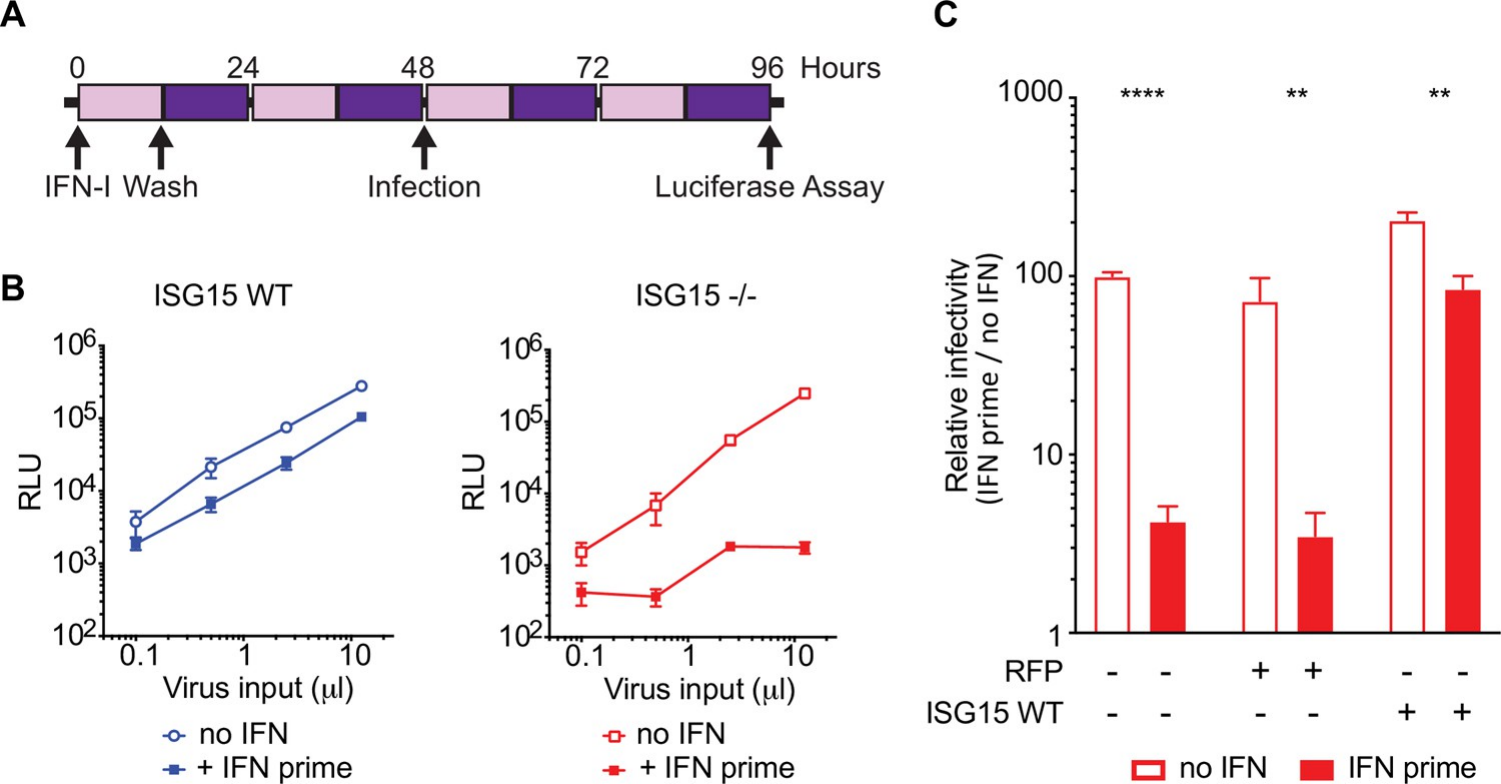
ISG15 deficient, IFN-primed cells are less susceptible to HIV-1 infection. A: hTERT-immortalized fibroblasts were primed with type I IFN (IFNα2b, 1000 units/mL) for 12 hours, washed and rested for 36 hours followed by infection with HIV-1-VSV-Luc. Cells were lysed 48 hours after infection, upon which HIV-1-luciferase expression was measured. B: Serially diluted HIV-1 (0.1, 0.5, 2.5 and 12.5 μL) was used to infect WT and ISG15-/- cells with and without IFN priming. Infections were done using cell lines from 2 unrelated WT controls and 2 unrelated ISG15-/- patients. Representative experiments are shown. RLU: relative light units. C: The parental ISG15-/- cell lines were complemented with RFP or ISG15 and infected as described above. Complementation with ISG15 abolishes the restriction. All infections were conducted in triplicates. Error bars represent SD. Significance was determined using t-tests (Prism 7 software; **** denotes P<0.0001; ** denotes P<0.01).

To determine HIV-1 susceptibility of ISG15-deficient, IFN-I-primed cells, we used hTERT-immortalized fibroblasts derived from ISG15-deficient patients to generate a tractable cell culture model system [7]. We used healthy control or ISG15-deficient patient fibroblasts transduced with either RFP-luciferase (negative control) or wildtype (WT) ISG15 [7]. Cells were prime-rested with IFN-I by treating them with 1000 IU/mL IFN_α2b_ for 12 hours, upon which cells were washed and allowed to rest for 36 hours (Fig 1A). At this timepoint, cells with WT ISG15 are no longer signaling an IFN-I response, whereas cells lacking ISG15 remain in an elevated inflammatory state, which recapitulates chronic IFN-I signaling that persists in patients with ISG15 deficiency [6]. After this prime-rest, cells were infected with increasing doses of a single-cycle HIV-1 NL4-3 Δenv expressing Firefly Luciferase in the position of Nef and pseudotyped with a VSVg envelope (HIV-1e^-^ Luc/VSVg). At day 2 post infection (96h in Fig 1A), the level of infection was measured by quantifying luciferase expression. Single-cycle HIV-1 infection was reduced by more than 100-fold in IFN-I-primed ISG15-deficient cells compared to unprimed ISG15-deficient cells, while only a modest 2.8-fold difference was observed in WT IFN-primed cells compared to WT unprimed cells (Fig 1B). This result suggests that, indeed, the absence of ISG15 limits HIV-1 infection.

To confirm that the effect observed was specifically dependent on ISG15 deficiency, we complemented the ISG15-deficient or healthy control cells with WT ISG15 or RFP as a negative control and infected these cells following the same prime-rest procedure outlined in Fig 1A. Consistent with Fig 1B (right), ISG15-deficient, IFN-I-primed cells are less susceptible when compared to ISG15-deficient unprimed cells (Fig 1C). Complementation of ISG15-deficient cell lines with WT ISG15 yields IFN-I-primed cells that are more susceptible to HIV-1 infection compared to RFP-complemented ISG15-deficient cells. Taken together, these data suggest that ISG15-deficient cells restrict HIV-1 in an ISG15-dependent manner.

### Complementation with WT or ΔGG ISG15 is sufficient to restore HIV-1 susceptibility

**Fig 2:**
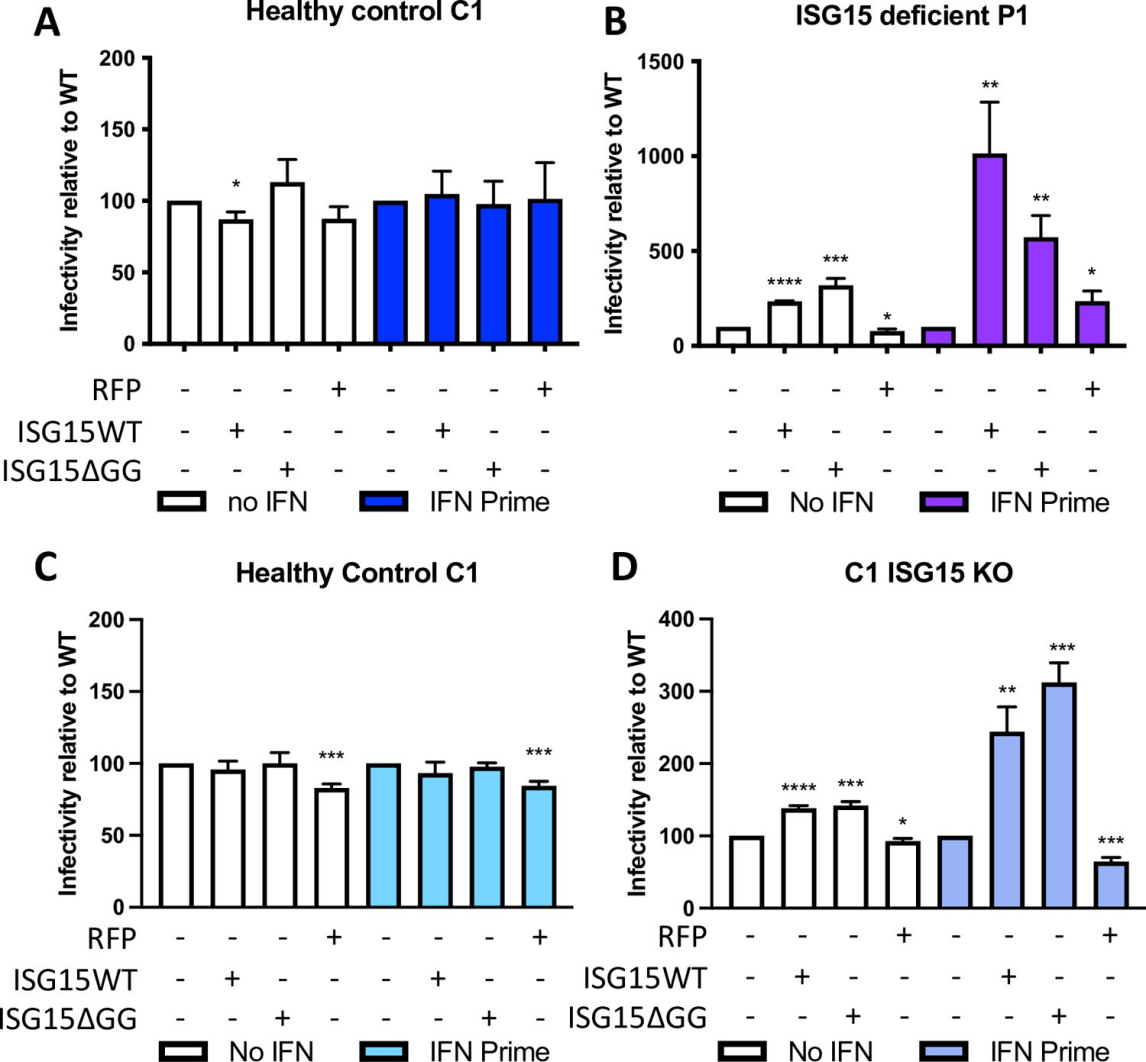
ISG15 complementation restores HIV-1 susceptibility. A, B,: hTERT-immortalized fibroblasts from healthy controls (A) and ISG15-deficient patients (B) were complemented with WT ISG15, ISGylation defective ISG15, or Luciferase RFP as a negative control. Cells stimulated with IFN-I overnight and rested for 36 hours were infected with HIV-1-VSV-GFP reporter virus. Flow cytometry was performed to quantify GFP-positive HIV-1-infected cells. Infectivity normalized to the parental cell-line is shown for mock primed (no IFN) and IFN primed cells. Error bars denote SD. (N = 4) **** denotes P<0.0001; *** denotes P<0.001; ** denotes P<0.01; * denotes P < 0.05 as determined by student’s two-tailed t-test. C, D: hTERTimmortalized fibroblasts from healthy controls (C) or ISG15 knockout healthy controls (D) were complemented with WT ISG15, ISGylation defective ISG15 (ISG15ΔGG), or Luciferase RFP as a negative control. Cells stimulated with IFN-I overnight and rested for 36 hours were infected with HIV-1-VSV-GFP reporter virus. Flow cytometry was performed to quantify GFP-positive HIV-1-infected cells. Infectivity normalized to the parental cell-line is shown for mock primed (no IFN-I) and IFN-I primed cells. Error bars denote SD. (N = 3) **** denotes P<0.0001; *** denotes P<0.001; ** denotes P<0.01; * denotes P<0.05 as determined by a student’s two-tailed T test.

To test whether ISGylation or the negative regulatory function of free ISG15 is necessary for the observed proviral phenotype, we used healthy control and ISG15-deficient patient-derived fibroblasts, transduced with either empty vector containing RFP-luciferase (negative control), WT ISG15, or ISG15ΔGG [7]. The ISG15ΔGG mutation was previously shown to maintain the negative regulatory capacity due to its retained ability to bind and stabilize USP18, while failing to modify targets by ISGylation [7]. Fibroblasts were prime-rested as previously described in Fig 1A and infected with single cycle HIV-1e^-^ GFP/VSVg (in this case, luciferase was replaced by GFP to allow for enumeration of infected cells by flow cytometry) as described above. GFP-positive cells were quantified by flow cytometry analysis, and infectivity was calculated as percent of GFP-positive cells relative to C1 healthy control cells (Fig 2A and 2B). As anticipated, ISG15 complementation of control cells, which endogenously express ISG15, did not change susceptibility to infection with or without IFN-I-priming (Fig 2A). Complementation with WT ISG15 in the ISG15-deficient patient cells significantly increased the percent of GFP-positive cells in IFN-I-primed cells (Fig 2B). Similarly, complementation with ISG15ΔGG in the ISG15-deficient patient cells also significantly increased infection in IFN-primed cells (Fig 2A and 2B), while complementation with RFP did not change the number of HIV-1 infected cells. To confirm these results in an isogenic system, we also compared an ISG15 CRISPR knock out (KO) cell line (C1 ISG15^KO^) generated on a WT background to their parental line (healthy control C1), by infecting each with single-cycle HIV-1e^-^ GFP/VSVg and quantifying infectivity by flow cytometry analysis on day 3 post infection (Fig 2C and 2D). Again, complementation with WT ISG15 or ISG15ΔGG in C1 ISG15^KO^ cells, but not control C1 cells, increased infection in IFN-I-primed cells. Together these findings indicate that the proviral role of ISG15 in HIV-1 infection is an ISGylation independent process.

### ISG15-deficient primary CD4+ T cells were less susceptible to HIV-1 infection

**Fig 3:**
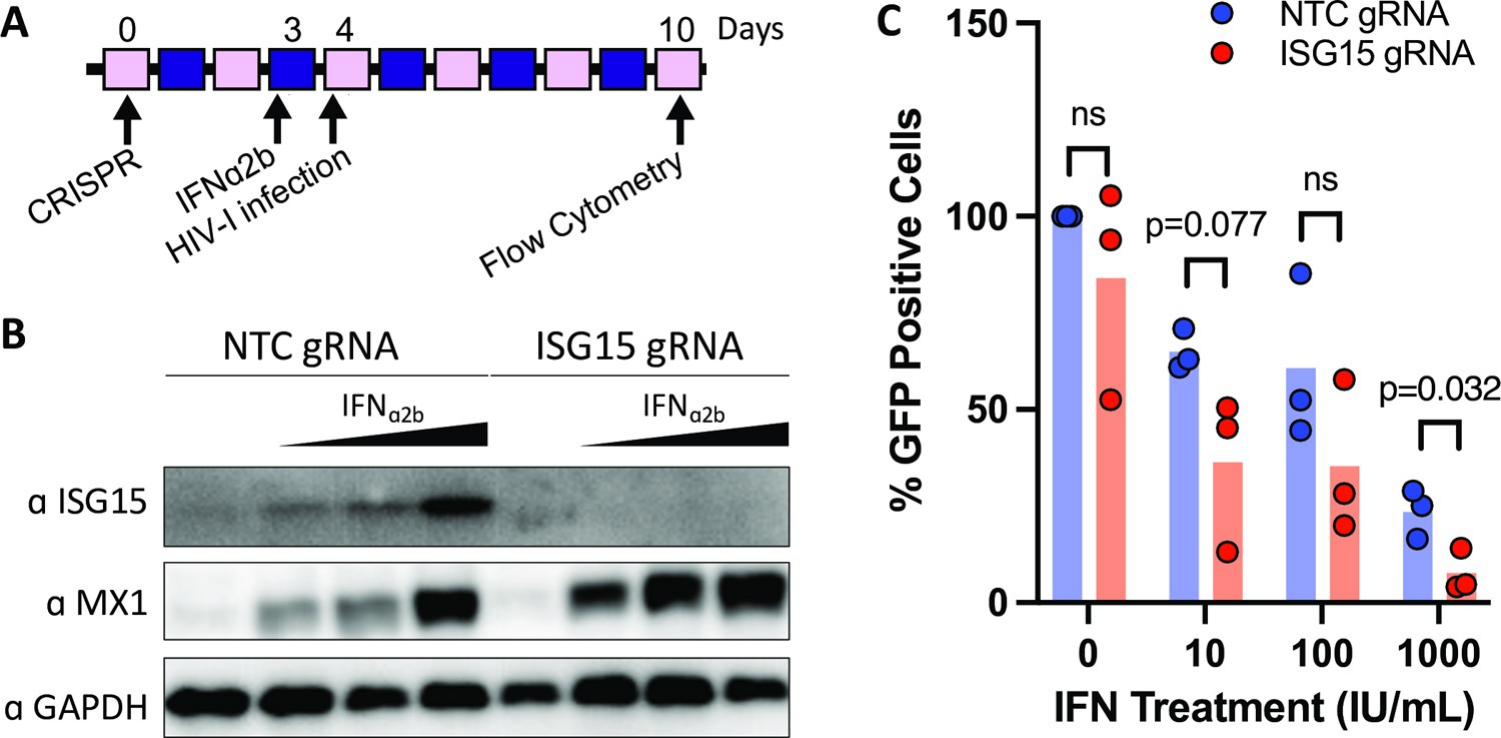
ISG15-deficient primary CD4+ T cells are less susceptible to HIV-1 infection. A: Primary CD4^+^ T cells were targeted with either non-targeting control (NTC gRNA) or ISG15 (ISG15 gRNA) CRISPR RNPs. On day 3, cells were treated with 0, 10, 100, or 1000 IU/mL IFNα2b for 24 hours. On day 4 (24 hours after IFN treatment) cells were infected with replication-competent HIV-1-GFP. Flow cytometry was performed on Day 10 (6 days post HIV-1 infection) to quantify GFP-positive HIV-1-infected cells. B: ISG15 knockout efficiency in primary human CD4^+^ T cells was assessed by Western blotting. Representative Western blot of CRISPR-targeted CD4^+^ T cells lysed 24 hours post IFNα2b treatment. C: Infection of IFNα2b-primed, CRISPR-targeted CD4^+^ T cells using HIV-1-GFP was performed in duplicate. Duplicates were averaged and percent infectivity was calculated relative to NTC-targeted non-IFN-I treated. Three donors are shown. GFP-positive cells were quantified 6 days post infection. Multiple, unpaired T tests were performed and P value is denoted in the figure. NS denotes T test result was non-significant.

To more closely approximate natural HIV-1 infection, we next investigated the role for ISG15 with and without an IFN-I prime in primary human CD4^+^ T cells, the physiological target of HIV-1 infection. We generated ISG15 knockout CD4^+^ T cells using CRISPR-Cas9 nucleofection (outlined in Fig 3A). CD4^+^ T cells were edited with either non-target control guide 1 (NTCg1) or ISG15 guide 1 (ISG15g1) CRISPR Cas9 ribonucleoproteins (RNPs). Efficient ISG15 knockout in primary CD4^+^ T cells (with and without 24 hours of IFN-I treatment) was confirmed by Western blot (Fig 3B). Four days after CRISPR genome editing (and 24 hours after IFN-I treatment), we observed a low level of basal ISG15 expression in NTCg1-targeted cells, which increased upon IFN-I treatment (Fig 3B, left). In contrast, ISG15g1-targeted cells did not express detectable levels of ISG15 protein with or without IFN-I treatment (Fig 3B, right).

In order to determine the role of ISG15 in HIV-1 infection in primary CD4^+^ T cells, we infected the ISG15^KO^ CD4^+^ T cells with HIV-1. Briefly, CRISPR-targeted cells that were stimulated with anti-CD3/CD28 were rested for 3 days, after which they were stimulated with 0, 10, 100, or 1000 IU/mL of IFNɑ2b (Fig 3A). Samples of cells were lysed for Western blot analysis to validate ISG15 knockout (Fig 3B). After 24 hours of IFN-I stimulation (day 4, Fig 3A), cells were infected with replication-competent HIV-1 NL4-3 Nef-IRES-GFP (HIV-1-GFP) for 8 hours. Infection was measured by quantification of GFP-positive cells by flow cytometry at day 6 post infection (day 10 after CRISPR, Fig 3A). Individual donor GFP-positive percentage results are shown in S1 Fig. To combine results of three donors, duplicates were averaged and percent infectivity was calculated relative to NTC-targeted cells without IFN-I treatment. Our results demonstrate that ISG15 KO cells are less susceptible to HIV infection when pretreated with 1000 IU/mL of IFNɑ2b (Fig 3C). This is true for the 10 and 100 IU/mL doses as well, but the donor variation limits the statistical significance.

Interestingly, MX1, an antiviral host protein, expression is increased in IFN-I stimulated, ISG15 gRNA targeted cells compared to NTC gRNA targeted controls (Fig 3B), suggesting ISG15-deficient cells upon IFN-I stimulation may be in a heightened antiviral state compared to control IFN-I stimulated cells. Taken together, the loss of ISG15 expression restricted HIV-1 infection, not only in hTERT-immortalized fibroblasts, but also in primary human CD4^+^ T cells, the main target cell population of HIV-1.

### Loss of ISG15 in primary CD4^+^ T cells lead to an increase in select interferon stimulated genes

To investigate the mechanisms of how ISG15 deficiency shapes the transcriptomic landscape to enhance HIV-1 immunity of T cells, we performed RNA sequencing on CRISPR-edited primary CD4^+^ T cells. Non-electroporated, NTCg1- and ISG15g1-targeted CD4^+^ T cells were incubated with IFNβ (1,000 IU/mL) for 24 hours and lysed prior to RNA extraction and RNA sequencing (RNAseq). Unstimulated non-electroporated, NTCg1- and ISG15g1-targeted CD4^+^ T cells were used as controls.

**Fig 4:**
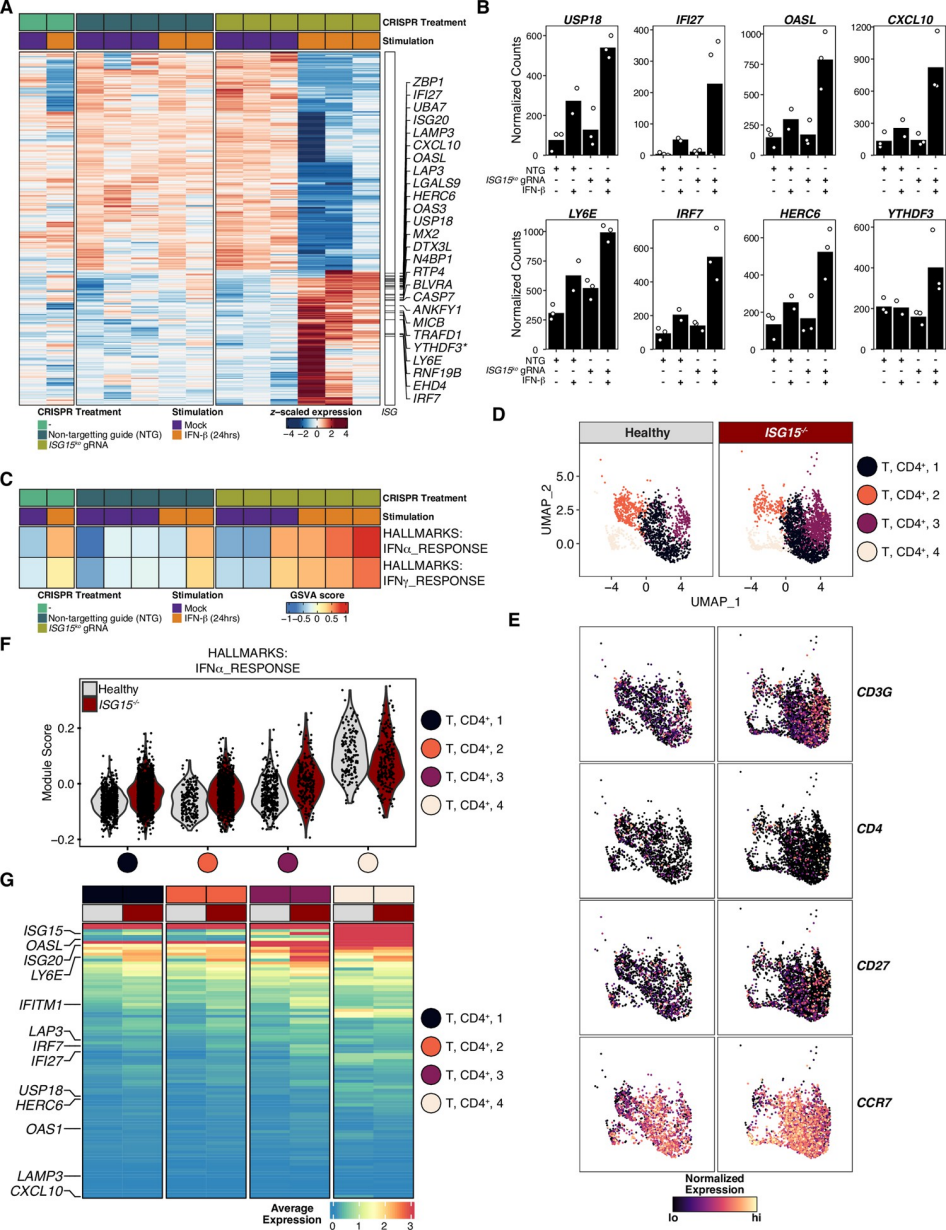
RNA-seq analysis of CRISPR-Cas9 ISG15 knockout in primary CD4^+^ T cells reveals parallels to human ISG15 deficiency. A: Differential gene expression heatmap between IFNβ stimulated (1000 IU/mL) CRISPR-Cas9 ISG15 knockout primary CD4^+^ T cells and non-targeting/non-edited controls shows elevated levels of interferon-stimulated genes (ISGs) in ISG15^ko^ primary CD4^+^ T cells. Represented genes are the union of differentially expressed genes identified during IFN_β_ stimulation of CRISPR-Cas9 ISG15^ko^ and IFNβ stimulation of the non-targeting controls, respectively. Differentially expressed genes (DEGs) were further filtered against a p-value of p<0.01. Flagged genes represent differentially expressed interferon-stimulated genes. YTHDF3 is not an ISG and is denoted by a star in the heatmap. B: Normalized count bar plots derived from RNA-seq data of select differentially expressed genes during IFN-b stimulation reveal elevated levels of ISGs in ISG15^ko^ primary CD4^+^ T cells relative to non-targeted guide control cells. Bars represent mean normalized count values. C: Gene set variation analysis of differentially expressed genes identified during IFN-b stimulation of CRISPR-Ca9 ISG15^ko^ primary CD4^+^ T cells confirms an IFN-I response signature in ISG15^ko^ primary CD4^+^ T cells. D: UMAP representation of unsupervised clustering results of single-cell RNA of 3,780 ISG15-/- patient (n = 2357 cells) and age-matched healthy control (n = 1423 cells) peripheral blood mononuclear cells. Points are colored by CD4^+^ T cell subset supervised annotation. E: Gene expression UMAPs of classical CD4^+^ T cell marker gene RNA transcripts in ISG15^-/-^ patient and age matched healthy control PBMCs. Point color intensity is scaled between the 1^st^ percentile (lo) and the 99^th^ percentile (hi) of natural log normalized gene expression count values. F: Gene set score distributions for IFN_α_ signaling in CD4^+^ T cell subsets. The HALLMARKS:IFNa_RESPONSE gene set was extracted from MSigDB Hallmark collection and used as input in Seurat’s AddModuleScore function. G: Average gene expression heatmap of the HALLMARKS: IFNa_RESPONSE gene set in ISG15^-/-^ patient and healthy control CD4^+^ T cell subsets. Heatmap color intensity represented log normalized average expression. Average expression value for each patient-cluster condition calculated from Seurat’s AverageExpression function.

The transcriptome analysis revealed that the knock-down of ISG15 in primary CD4^+^ T cells resulted in upregulation of a specific ISG signature upon IFNβ stimulation (Fig 4A and 4B). Among these, *IFI27*, *ISG20*, *HERC6*, *MX2*, *N4BP1*, *MICB*, *YTHDF3*, *and IRF7* have previously been described to negatively affect HIV-1 replication, [42–44,58–63]. In addition, our study also indicated a putative role for novel mediators of HIV-1 immunity, namely *ZBP1*, *LAMP3*, *CXCL10*, *LGALS9*, and *ANKYF1*, all of which have been associated with HIV-1/AIDS disease progression [64–71]. LY6E was shown to differentially regulate HIV-1 infection depending on cell surface CD4 expression levels [71,72]. Although not a canonical ISG, we document YTHDF3 expression as increased in ISG15-deficient CD4^+^ T cells stimulated with IFN-I (Fig 4A). We and others previously reported that YTHDF3 negatively affects HIV-1 replication by limiting HIV-1 reverse transcription [62,73]. Further, unsupervised gene set variation analysis showed expected expression signatures in IFNβ stimulated primary CD4^+^ T cells (Fig 4C). Together, these results suggest that there is likely a set of ISGs in ISG15-deficient CD4^+^ T cells that is sufficient to reduce the susceptibility of primary CD4^+^ T cells to HIV-1 infection.

To assess whether the elevated ISG expression signatures detected were comparable to patterns identified in ISG15-deficient patients, we re-analyzed a single cell RNA-sequencing dataset [34,74] of PBMCs isolated from an ISG15-deficient patient and a healthy control (Fig 4D). A closer investigation into the CD4^+^ T-cell compartment (defined based on the expression of canonical markers), our supervised analysis identifies four clusters present in both ISG15-deficient patient and healthy controls (Fig 4D and 4E). Gene set score analysis of the IFN-alpha response signature at single cell resolution reveals elevated levels of ISGs in ISG15-deficient patient CD4^+^ T cell subsets, with significant differences in clusters 1 and 3 (Fig 4F). Evaluation of average expression values of IFN-I related genes in the CD4^+^ T cell compartment suggests that our CRISPR edited ISG15^KO^ primary CD4^+^ T cell model are transcriptionally similar to patient derived CD4^+^ T cells (Fig 4G). Indeed, the transcriptional program of CD4^+^ T cells from an ISG15-deficient patient noted here is likely to result in increased resistance to HIV-1 replication.

## Discussion

The function of ISG15 in HIV-1 immunity remains an area of active investigation [6,8,36]. We observed here that ISG15-deficient patient fibroblasts were more resistant to HIV-1 infection than WT cells (Figs 1 and 2). This susceptibility was reversed when cells were complemented with WT or ΔGG ISG15 (Figs 1 and 2), suggesting that ISGylation was dispensable for this process. We found ISG15 deficiency limits HIV-1 infection not only in fibroblasts, but also in human primary CD4^+^ T cells (Fig 3).

Our results point to ISG15 as a predominantly positive regulator of HIV-1 infection (“a proviral ISG”) in human cells, unlike previous studies suggesting an antiviral role for ISGylation. It was previously reported that ISG15 expression is elevated in PBMCs of patients infected with HIV-1 compared to healthy controls, and that ISG15 expression levels correlated with higher plasma viral loads [75]. While this elevation is likely a result of the elevated systemic inflammation in HIV-1 positive individuals, these elevated levels of ISG15 may be advantageous for HIV-1 replication as it enhances the inhibition of IFN-I signaling.

Since IFN-I pre-treatment limited HIV-1 infection in WT primary human CD4^+^ T cells (Fig 3C and 3D), and to a greater extent in ISG15^KO^ cells, upregulation of a combination of differentially expressed genes is likely responsible for the HIV-1 resistance demonstrated here. Of the upregulated genes, a large fraction are ISGs (Fig 4A, right). Among these ISGs, many of them have previously been reported to be antiviral against HIV-1 or antiviral in general and likely contribute to limiting HIV-1 replication in the absence of ISG15 (Fig 4A–4C).

The results of this study align with previous results demonstrating that ISG15 deficiency restricts different viral infections [6–8,25,34–36]. Interestingly, ISG15 has previously been shown to act as an antiviral HIV-1 restriction factor. First, ISGylation was shown to restrict HIV-1 by limiting accumulation of misfolded p53 [56]. This manuscript demonstrates that the protease activity of USP18 stabilizes misfolded mutated p53, which requires ISG15 for its degradation. Second, ISGylation of Gag by the E3 ligase HERC5 was shown to restrict HIV-1 particle production in U2OS cells [54]. Finally, overexpression of ISG15 was shown to restrict HIV release in 293T cells [53]. While all these well controlled studies indicate that ISG15 can be antiviral, these studies did not examine the role for ISG15 in the context of its negative regulatory capacity of IFN-I. In the naturally occurring ISG15 deficiency, the primary role for ISG15 is that of a negative regulator of IFN-I, which minimizes ISG15’s function as an antiviral molecule.

Furthermore, and in line with our work, two independent studies show that USP18 knockdown restricts HIV replication [52,57]. Given that ISG15 acts to stabilize USP18, which negatively regulates JAK-STAT signaling, this makes ISG15 deficiency a *de facto* partial USP18 deficiency [7], which results in restriction of HIV-1 replication.

Overall, our findings confirm the principal role for ISG15 as a negative regulator of IFN-I signaling and points to novel ISGs in primary human CD4^+^ T cells whose effector function in the context of HIV-1 replication remains to be explored.

## Materials and methods

### Ethics statement

Peripheral blood lymphocytes were purchased from New York Blood Center from anonymous donors. The investigators had no direct interactions with blood donors or influence on the selection of PBMCs. This work is regarded as non-human subject research.

### Cell culture

hTERT immortalized fibroblasts were stably complemented with ISG15, ISG15ΔGG or luciferase by lentiviral transduction [6,7]. hTERT immortalized fibroblasts, and the TZM-bl reporter cell-line (cat#8129, NIH AIDS Reagent Program, Division of AIDS, NIAID, National Institutes of Health [76–80]) were maintained in Dulbecco’s modified Eagle medium (DMEM, Corning) in the presence of 10% fetal bovine serum (FBS; GemCell), 100 IU penicillin, and 100 μg/mL streptomycin (D-10) at 37°C and 5% CO_2_.

Primary human CD4^+^ T cells were purified from peripheral blood lymphocytes obtained from anonymous healthy blood donors (New York Blood Center). Ficoll (Ficoll Hystopaque; Sigma) density centrifugation was performed as per the manufacturer’s instructions. CD4^+^ T cells were negatively selected using magnetic beads (CD4^+^ T-cell Isolation Kit I; Miltenyi Biotec) and were maintained in RPMI 1640 (Corning) supplemented with 10% FBS (Gibco), 100 IU penicillin, 100 μg/mL streptomycin, 1x Glutamax, 10 mM HEPES, and 20 U/mL recombinant human IL-2 (NIH AIDS Reagent Program, Division of AIDS, NIAID, National Institutes of Health) (R-10-IL2) at 37°C and 5% CO_2_.

### Genome editing

Primary human CD4^+^ T cell Cas9 Ribonucleoprotein (RNP)-mediated gene editing experiments were carried out as previously described [62,81,82]. Primary CD4^+^ T cells (2.5 × 10^6^ cells/mL) were stimulated for 3 days at a volume of 500 μL per well with plate-bound anti-CD3 (Clone UCHT1; Tonbo) and suspended anti-CD28 (5 μg/mL; Clone CD28.2; Tonbo). Guide RNA (gRNA) was designed using the Benchling tool to target ISG15 (ISG15g1: 5’-CCGCCAGCATCTTCACCGTC-3’). The non-targeting control guide (NTCg1) used was previously published [82]. Generation of Cas9 RNPs and nucleofection of primary CD4^+^ T cells was performed exactly as previously described [62].

### Western blots

Primary CD4^+^ T cells were lysed in RIPA buffer (Thermo Fisher Scientific) supplemented with 1x protease/phosphatase inhibitor cocktail (Cell Signaling Technology). Lysates were incubated with DTT and NuPAGE LDS sample buffer (Invitrogen). Lysates were run on 12% Criterion TGX gels (BioRad) and transferred to Amersham Hybond polyvinylidene difluoride (PVDF) blotting membranes (Cytiva). Western blots were blocked in 5% BSA in TBS 0.1% Tween and washed in TBS 0.2% Tween. Western blots were incubated with secondary antibody in 5% milk in TBS 0.1% Tween. Western blot chemiluminescence was detected with SuperSignal^TM^ West Femto Substrate (Thermo Scientific). Imaging of the Western blot bands was performed using AlphaView software (ProteinSimple). The following antibodies were used for immunoblots: α-ISG15 (clone F9, 1:500, Santa Cruz Biotech, Cat# sc-166755), α-MX1 (1:1000, Abcam, ab95926), α-GAPDH (clone 6C5, 1:10,000, Sigma Aldrich, MAB374).

### Plasmids

The HIV-1 NL4-3 Δenv Luc construct was kindly provided by N. R. Landau [83]. HIV-1 NL4-3 Δenv eGFP (reagent #11100, [84]) was obtained through the AIDS Research and Reference Reagent Program, Division of AIDS, NIAID, National Institutes of Health. The VSVg envelope was expressed from plasmid phCMV G [85]. The HIV-1 NL4-3 Nef-IRES-GFP (HIV-1-GFP) construct was kindly provided by B. K. Chen [86].

### Production of viral stocks

Single-cycle viruses HIV-1 NL4-3 Δenv-eGFP and HIV-1 NL4-3 Δenv-Luc were generated and pseudotyped with VSVg by transfection of HEK293T cells with Polyethylenimine (PEI) (Polysciences). Three days after transfection, culture supernatants were harvested, clarified at 500 x G, filtered (0.45 μm), aliquoted, and stored at -80°C.

Replication competent (HIV-1-GFP) virus was generated by transfection of HEK293T cells with PEI (Polysciences). Two days after transfection, culture supernatants were harvested, filtered (0.45 μm), aliquoted, and stored at -80°C.

TZM-bl cells containing the β-galactosidase reporter gene driven by the HIV-1 long terminal repeat, were used to determine viral titers (TCID50/mL) of viral stocks produced as previously described [87].

### IFN-I priming

Fibroblasts were prime-rested as described previously [6], by treating with IFNα2b (Merck Intron A 0085-4350-01) (1000 IU/mL) in D-10 media for 12 hours, upon which cells were washed three times with PBS, fresh D-10 media was added, and the cells were allowed to rest for 36 hours before infection.

CRISPR-targeted CD4^+^ T cells were stimulated with IFNɑ2b (10, 100, 1000 IU/mL) in R-10-IL2 media for 24 hours.

### HIV-1 infection experiments

Prime-rested fibroblasts were infected with increasing doses of HIV-1-VSV-Luc. The level of infection was determined 48 hours post infection by quantifying Firefly Luciferase using the Luciferase Assay System kit (Promega).

Prime-rested fibroblasts were infected in triplicate with HIV-1-VSV-GFP overnight in the presence of polybrene (5 μg/mL). D-10 media was changed the next morning. The level of infection was determined on day 4 post infection by quantifying GFP-positive fibroblasts by flow cytometry (Luminex Guava easyCyte). Flow cytometry data was analyzed with Luminex Guava InCyte software.

IFN-primed CD4^+^ T cells were infected with HIV-1-GFP for 8 hours in the presence of polybrene (2 μg/mL). After 8 hours, viral supernatant was removed and R-10-IL2 culture media was replenished. Media was changed on days 3 and 5 post infection. The level of infection was determined on day 6 post infection by quantifying GFP-positive CD4^+^ T cells by flow cytometry (Luminex Guava easyCyte). Flow cytometry data was analyzed with Luminex Guava InCyte software.

### RNAseq

Primary CD4^+^ T cells were nucleofected with NTCg1 or ISG15 CRISPR RNPs as described above for 3 days and then either stimulated or not with 1000 U/mL of IFNβ1b (PBL Assay Science, cat. 11420–1) for 24h. Cells were lysed in TRIzol Reagent (Invitrogen) and RNA was extracted per the manufacturer’s instructions. Total RNA was treated with RNase-Free DNase (QIAGEN 79254) and prepped for RNAseq with the TruSeq RNA Sample Prep Kit v2 (Illumina). 75 bp unpaired reads were generated on a NextSeq 550 (Illumina). Raw bcl image processing, fastq generation, reference genome alignment and feature counting were conducted using the RNA Express module in the BaseSpace suite (Illumina). Raw gene-sample counts matrices were extracted and read into the R statistical environment and further analyzed using the DESeq2 package [88]. Differentially gene expression testing was conducted contrasting CRISPR ISG15^ko^ primary CD4^+^ T cells during IFN stimulation. Similar DGE testing was done contrasting the non-targeting guide controls. Resulting gene lists were filtered on a p-value of p<0.01 and expression values visualized using the ComplexHeatmap and ggplot2 packages.

### Single-cell RNAseq (scRNAseq)

Previously published single-cell RNA-sequencing data of PBMCs from a patient with human ISG15 deficiency (N = 1) and a healthy control (N = 1) from Martin-Fernandez et al. (34) was accessed and analyzed. Raw gene-cell matrices were read into the R (v4.0.4) statistical environment and analyzed using Seurat (v4.0.1) for quality control, integration, clustering, and differential gene expression [89]. For quality control, data were filtered to exclude genes detected in less than three cells (per subject), to exclude cells with < 200 expressed genes (empty droplets) and to exclude cells with > 7.5% UMIs assigned to mitochondrial genes (dying cells). Filtered data were independently normalized using Seurat’s SCTransform function (developer’s default parameters) [90]. To account for subject-specific effects, both data sets were integrated using Seurat’s FindIntegrationAnchors and IntegrateData functions (developer’s default parameters). Dimensional reduction of the integrated data set was performed by principal component analysis (PCA) and first 25 principal components were used for unsupervised graph-based clustering (resolution: 1.2) and visualized by Uniform manifold approximation and projection (UMAP; parameters: n.dims: 25, n.neighbors: 30, metric: cosine). Supervised analysis was then conducted to subset CD4+ T cell clusters using canon immune markers (CD3, CD4, CD27, CCR7). IFNa gene signature scores (input genes were included from MSigDB HALLMARKS: IFNa RESPONSE) were then computed at single-cell resolution using Seurat’s AddModuleScore function.

## Supporting information

S1 Fig Primary CD4^+^ T cells were targeted with either non-targeting control (NTC gRNA) or ISG15 (ISG15 gRNA) CRISPR RNPs. *On day 3*, *cells were treated with 0*, *10*, *100*, *or 1000 IU/mL IFN*_α2b_ for 24 hours. *On day 4 (24 hours after IFN treatment) cells were infected with replication-competent HIV-1-GFP*. *Infection of IFN*_α2b_*-primed*, *CRISPR-targeted CD4*^*+*^ *T cells using HIV-1-GFP was performed in duplicate*. *Flow cytometry was performed on day 6 post infection to quantify GFP positive cells*. *The percent of GFP positive cells is shown for three donors*.
(TIF)
