# Supplementary figures and images for: ISG15 deficiency restricts HIV-1 infection
Source: PLoS Pathog. 2022 Mar 25;18(3):e1010405. doi: 10.1371/journal.ppat.1010405 (PMC8986114; doi:10.1371/journal.ppat.1010405)

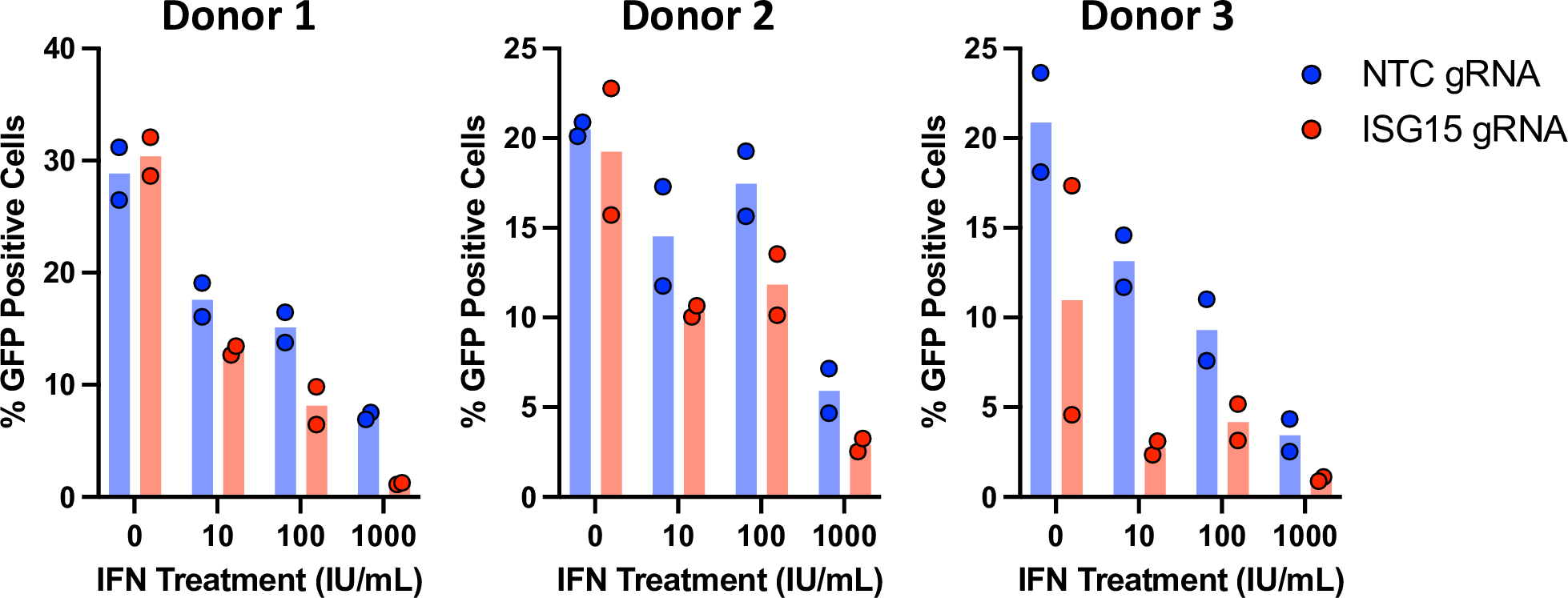

Supplement: S1 Fig — On day 3, cells were treated with 0, 10, 100, or 1000 IU/mL IFNα2b for 24 hours. On day 4 (24 hours after IFN treatment) cells were infected with replication-competent HIV-1-GFP. Infection of IFNα2b-primed, CRISPR-targeted CD4+ T cells using HIV-1-GFP was performed in duplicate. Flow cytometry was performed on day 6 post infection to quantify GFP positive cells. The percent of GFP positive cells is shown for three donors. (TIF) [file ppat.1010405.s001.tif]
